# Analytical procedures and methods validation for oxalate content estimation

**DOI:** 10.33263/briac95.305310

**Published:** 2019-09-18

**Authors:** Dina Karamad, Kianoush Khosravi-Darani, Hedayat Hosseini, Sanaz Tavasoli

**Affiliations:** 1 Faculty of Nutrition Sciences and Food Technology, National Nutrition and Food Technology Research Institute, Shahid Beheshti University of Medical Sciences, P.O. Box: 19395-4741, Tehran, Iran; 2 Research Department of Food Technology Research, National Nutrition and Food Technology Research Institute, Shahid Beheshti University of Medical Sciences, P.O. Box: 19395-4741, Tehran, Iran; 3 Department of Food Technology Research, National Nutrition and Food Technology Research Institute, Shahid Beheshti University of Medical Sciences, P.O. Box: 19395-4741, Tehran, Iran; 4 Urology and Nephrology Research Center, Shahid Labbafinejad Medical Center, Shahid Beheshti University of Medical Sciences, Tehran, Iran

**Keywords:** Enzymatic method, Method validation, NaOH, Oxalate measurements, Potassium permanganate, Titration

## Abstract

Increased urinary oxalate is considered a major risk factor in the formation of calcium oxalate kidney stones. Gut microbiota may reduce the risk of stone formation. Anyway, the first step for any research about monitoring of oxalate content (both in vitro and in vivo) is a determination of its concentration, while there are different methods reported in the literature for oxalate content determination. In this research, the main reported methods including titration with two titrators (potassium permanganate, and NaOH) as well as enzymatic method (oxalate assay kit) are presented and compared for the measurement of oxalate in both inoculated and non-inoculated media.

## 1. INTRODUCTION

Oxalic acid is a dicarboxylic acid found in all kinds of life including microorganisms, plants, and animals [22]. The content of oxalate in the human body depends on dietary intake (principally in seeds and leafy plants related to spinach and rhubarb) and the rate of synthesis via metabolism of ascorbic acid and glyoxylate in the liver. Dietary intake of a large amount of oxalate could be harmful and leads to the oxalosis or formation of calcium oxalate deposits in vital tissues or organs of the body [1,23,24,28]. The patients who are high risk for kidney stone problems should control oxalate intake to less than 40–50 mg/day (by the American Dietetic Association, 2005).

Gut bacterial flora can reduce the oxalate content of digested food through the enzymatic pathway [25–27]. *Oxalobacter* (*O.*) *formigenes* and *Lactobacillus* and *Bifidobact-erium* sp. are the main gastrointestinal (GI) flora with oxalate degradation [29,30,32]. *O. formigenes* degrades oxalate as its energy and carbon source within the GI tract of its hosts, in contrast to other known oxalate-degrading bacteria, nearly all of which only metabolize oxalate using detoxification pathways under specific conditions [33–35].

Measurement of oxalate content in foods is very important from the health aspects of human society [36]. The methods for determination of oxalate are titration [6], capillary electrophoresis [20], gas chromatography [15], and enzymatic analysis [11]. Anyway, some of the research about oxalate monitoring is based on the application of beneficial microorganisms which can colonize in the GI tract and reduce oxalate content. Some of the reported analytical methods cannot be applicable for microbiological investigation where some bacteria exist in the medium. The method of oxalate estimation by titration with NaOH is based on oxalic acid reaction with NaOH, while bacteria may produce other different organic acids via their metabolism pathways like propionic and lactic acid [2–4,12]. Application of these methods for inoculated and noninoculated media are not compared in previous research.

In this report, methods for oxalate determination including titration with potassium permanganate and NaOH as well as enzymatic method have been successfully used to measure oxalate content of samples. Then reproducibility of methods and their precision are compared.

## 2. MATERIALS AND METHODS

### 2.1. Material

All chemicals were of analytical grade and were purchased from Merck and apart from Sucrose, Sodium oxalate and di-Ammonium hydrogen-citrate which were purchased from Carlo company.

### 2.2. Sample preparation

Culture media containing 5, 10, 15 and 20 mmol/L sodium oxalate (MERK; Darmstadt, Germany) was prepared by adding 10 mL of the 0.46 μ filter sterilized sugar and ammonium oxalate solutions listed below to 10 mL base media proteose peptone no. 3 (MERK; Darmstadt, Germany), 10 g yeast extract (MERK; Darmstadt, Germany), 5 g Tween 80 (MERK; Darmstadt, Germany) 1 mL KH_2_PO_4_ (MERK; Darmstadt, Germany) 2 g Na acetate (MERK; Darmstadt, Germany) 5 g di-Ammonium hydrogen-citrate (MERK; Darmstadt, Germany) 2 g MgSO_4_.7H_2_O (MERK; Darmstadt, Germany) 0.05 g MnSO_4_ (Merck) 0.05 g water to 500 mL and sterilized at 121°C for 15 min.

For the preparation of 5, 10, 15 and 20 mmol/L sodium oxalate solutions, 13.39 g of Na_2_C_2_O_4_ (MW =133.96) transferred to a 100mL volumetric flask. Rinsed the boat into the flask through a funnel until the volume reaches to 100ml. It may need to heat this gently (NOT BOIL) to promote the dissolving. Then before autoclave sterilization of base media samples, a specified volume of sodium oxalate solution was added to the samples. The amount of sodium oxalate was derived from Eq 1:

Eq. 1 $${\text{C}}_{1}{\text{V}}_{1}={\text{C}}_{2}{\text{V}}_{2}$$

C_1_V_1_ = Concentration/amount (start) and Volume (start)

C_2_V_2_ = Concentration/amount (final) and Volume (final)

The required volume of sodium oxalate solution with (20 mmol/L concentration) to the 10 ml base media derived from Eq. 1.

### 2.3. Microbial assay

For investigation on biodegradation effect of bacteria on oxalate content, the *L. acidophilus* and *L. plantarum* were obtained from IBRS culture collection. The microorganisms were cultured in MRS broth [2]. The bacteria were cultured overnight at 37°C.

Then sample media inoculated at 10% with a fresh culture of *L. acidophilus* and *L. plantarum*, separately. To compare the validity and applicability of each considered methods the samples were divided to three vials: 1) for analyzing the solution before inoculation 2) analyzing after inoculation with *L. acidophilus*, 3) analyzing after inoculation with *L. plantarum*. Table 1 shows the details of contents of vials for titration by potassium permanganate

### 2.4. Titration with potassium permanganate

The technique of titration has been used previously in acid-base reactions to detect the amount of acid using a known base (or the reverse). It can also be used in situations in which the reaction involves oxidation and reduction.

Oxidation is defined as the loss of electrons (on the right of arrow) or increases in oxidation state (each C changes from +3 to +4) as shown below by the half-reaction involving oxalate ion.

$${\text{C}}_{2}{{\text{O}}_{4}}^{2-}\to 2\;{\text{CO}}_{2}+2{\text{e}}^{-}$$

The reduction is shown by the permanganate ion as it gains electrons and Mn decreases in oxidation state from +7 to +2.

$$8\;{\text{H}}^{+}+{{\text{MnO}}_{4}}^{-}+5\;{\text{e}}^{-}\to {\text{Mn}}^{2+}+4\;{\text{H}}_{2}\text{O}$$

Oxidation and reduction must occur together and are often designated as redox processes to emphasize this; the number of electrons lost by one substance must equal the number of electrons gained by the other substance. If we combine the two half-reactions above, we would end with a balanced net ionic equation if we have a total of 10 electrons exchanged.

$$16\;{\text{H}}^{+}+2\;{{\text{MnO}}_{4}}^{-}+5\;{\text{C}}_{2}{{\text{O}}_{4}}^{2-}\to 2{\text{Mn}}^{2+}+10\;{\text{CO}}_{2}+8\;{\text{H}}_{2}\text{O}$$

Because the materials we weigh and measure occur as compounds, it is often useful to have the balanced molecular equation (sometimes called the balanced total equation).

$$8\;{\text{H}}_{2}{\text{SO}}_{4}+2\;{\text{KMnO}}_{4}+5\;{\text{Na}}_{2}{\text{C}}_{2}{\text{O}}_{4}\to 2\;{\text{MnSO}}_{4}+10\;{\text{CO}}_{2}+{\text{K}}_{2}{\text{SO}}_{4}+5\;{\text{Na}}_{2}{\text{SO}}_{4}+8\;{\text{H}}_{2}\text{O}$$

Potassium permanganate is reduced because it contains the permanganate ion; we can also say that it behaves as an oxidizing agent because it causes something else to become oxidized (the oxalate). Sodium oxalate is oxidized and we could also specify it as the reducing agent because it causes the permanganate to become reduced. It can also be seen from both the net ionic equation or molecular equation that acid is required for this reaction to occur; i.e., H+ or H_2_SO_4_ show up in the balanced equations. We also know from the previous experience that a temperature higher than room temperature makes a reaction more rapid.

By reduction of MnO_4_^−^ (with an intense dark purple color) to the colorless ion of Mn^+2^, the color solution will turn to a faint pink color at the equivalence point. In the oxalate measurement method, permanganate will be reduced by oxalate, C_2_O_4_^2−^ in acidic conditions. Oxalate reacts very slowly at room temperature so the solutions are titrated hot to make the procedure practical. The solution is titrated hot to speed up the very slow reaction of oxalate at room temperature [16].

About 3 g of the potassium permanganates were added to clean 500-mL (or larger) flask containing 100 mL distilled water; swirl and mix well Then another 100-mL of distilled water was added, mix and swirl; it was continued until a total of 400 mL of distilled water has been added. This solution was too dark to find out whether all of KMnO_4_ has dissolved. So, by carefully transferring of liquid into another large container undissolved solid will left in the original flask, and should be solved with excess stirring and swirling. A 50-mL cleaned burette rinsed with distilled water and then with 10-mL of potassium permanganates solution. Then the burette was filled with the solution, making sure that the tip contains no air bubbles. After acidifying sample with 10 ml sulfuric acid, it warmed to 70–90°C. A couple of drops of phenol phetaleine was added and then titrated to a very faint pink endpoint. The final reading of KMnO_4_ in the burette has been recorded.

### 2.5. Titration with NaOH

In this method oxalic acid is determined by titration with NaOH solution. The purpose of an acid- base titration is to determine the concentration of the acidic solution by titrating it with a basic solution of known concentration until neutralization occurs [21].

Two g sodium hydroxide was added to a clean 250-mL volumetric flask and 100 mL of distilled water was added; swirl and mix well. As mentioned in the previous method, another 100-mL of distilled water was added, mix and swirl; it was continued until a total of 250 mL of distilled water has been added and then stirred and swirled. Then the cleaned (rinsed with distilled water and NaOH) burette was filled with the solution. After acidification of solution with a 10ml sulfuric acid, 2–3 drops of phenol phetaleine was added and titrated to a very faint pink endpoint. The final reading of KMnO_4_ in the burette has been recorded.

### 2.6. Enzymatic method (oxalate assay kit)

Oxalate Assay kit as a colorimetric method is a sensitive, easy-to-use, and high throughput adaptable kit for estimation of oxalate content in solution. In this assay, oxalate reacts with oxalate converter and oxalate enzyme mix to form an intermediate, which in turn visible in spectrophotometry. The assay kit can detect oxalate levels in the range of 0.05–0.7 mmol.

In this research oxalate kit was purchased from Darman Faraz Kave, (Tehran, Iran) [11]. Spectrophotometry device (OPTIMA, TOKYO, JAPAN) was used at OD=590 nm. Table 2 shows material supplied for oxalate assay kit.

### 2.7. Lyophilized coloring reagent preparation

A small amount of buffer solution should be added to coloring reagent vial and shake well.

### 2.8. Lyophilized enzymatic reagent preparation

1 ml of distilled water was added to each vial of lyophilized enzymatic reagent, mixed and kept 5 min at room temperature.

### 2.9. Kit Sample preparation

1 ml of sample was added to 1 ml of dilution solution and two spoons of active caracole were added to this solution. After 5 min shaking of the solution, it was centrifuge 10 min in 3000 g. Then 50 μL from the clear layer was sampled. Then 3 tubes with the mentioned structure in Table 3 were prepared.

These mixtures were gently mixed and kept at 37°C for 10 min. Then the absorbance of T and S tubes should be read in front of tube B at 590 nm wavelength. Calculation of oxalate concentration obtained from Eq.2. Oxalate concentration (mmol/L)=

Eq.2 $$\frac{Abs.T}{Abs.S}*0.25*2*\mathrm{sample}\;\mathrm{dillution}\;\mathrm{coefficient}$$

### 2.10. Statistical analysis

All data are presented as the mean±SD. The relationship between variables was tested by linear regression. A *P* value of less than 0.05 was considered significant.

## 3. RESULTS

**Figure 1.**
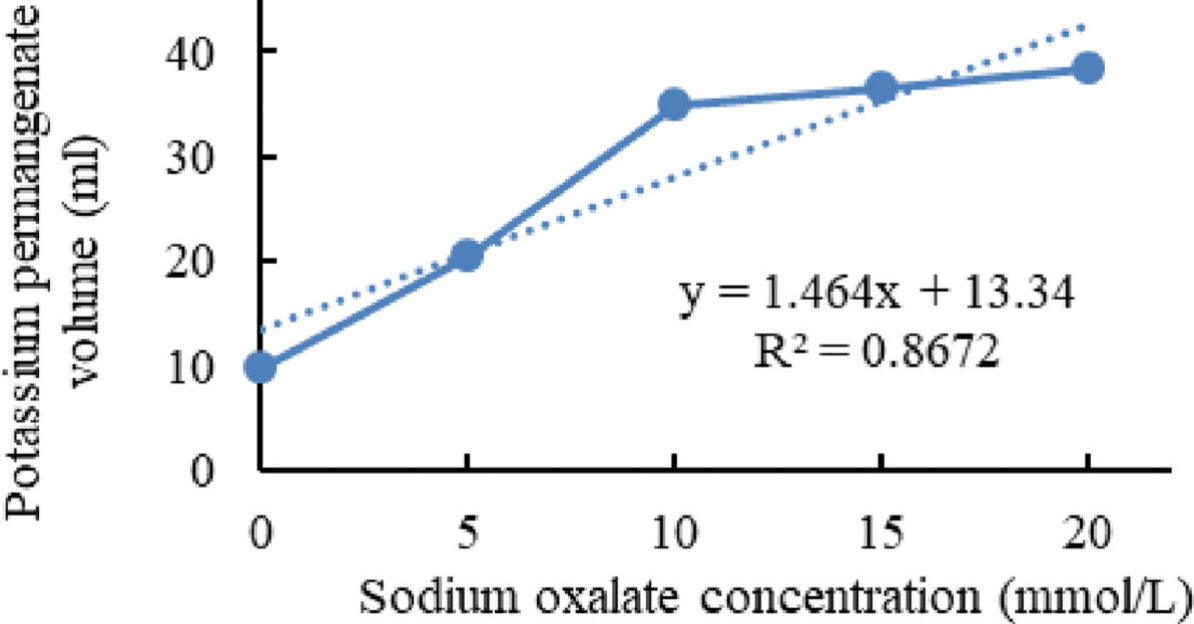
Calibration curves of sodium oxalate concentration and volumetric titration with potassium permanganate before inoculation.

**Figure 2.**
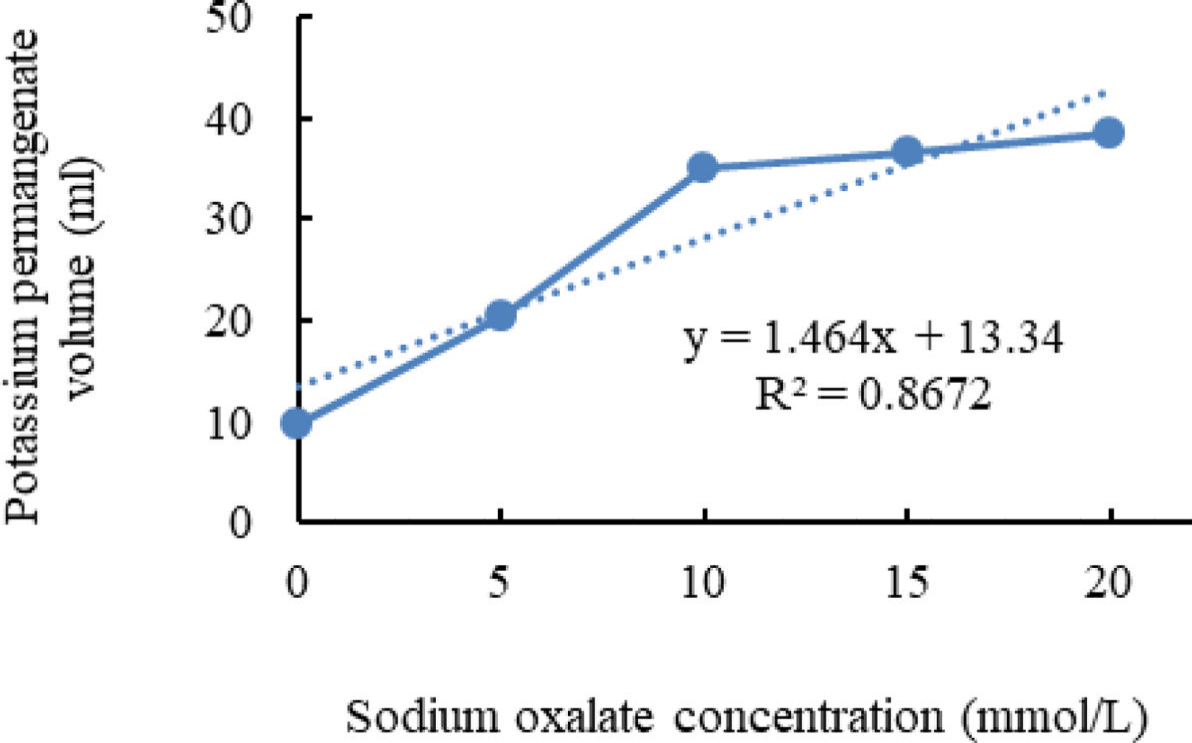
Calibration curves of sodium oxalate concentration and volumetric titration with potassium permanganate after inoculation with *L. acidophilus*.

**Figure 3.**
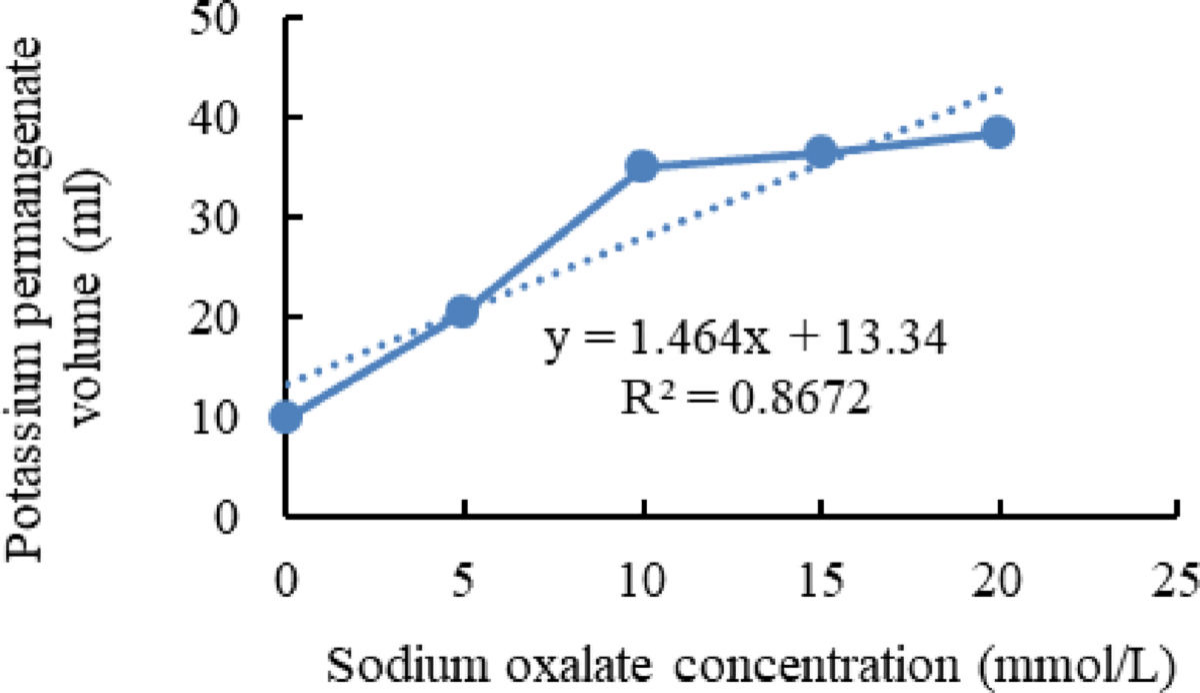
Calibration curves of sodium oxalate concentration and volumetric titration with potassium permanganate after inoculation with *L.plantarum*.

Figure 1–3 shows calibration curves of sodium oxalate concentration and volumetric titration with potassium permanganate before inoculation, after inoculation with *L. acidophilus*, and after inoculation with *L. plantarum*, respectively.

### 3.1. NaOH titration

**Figure 4.**
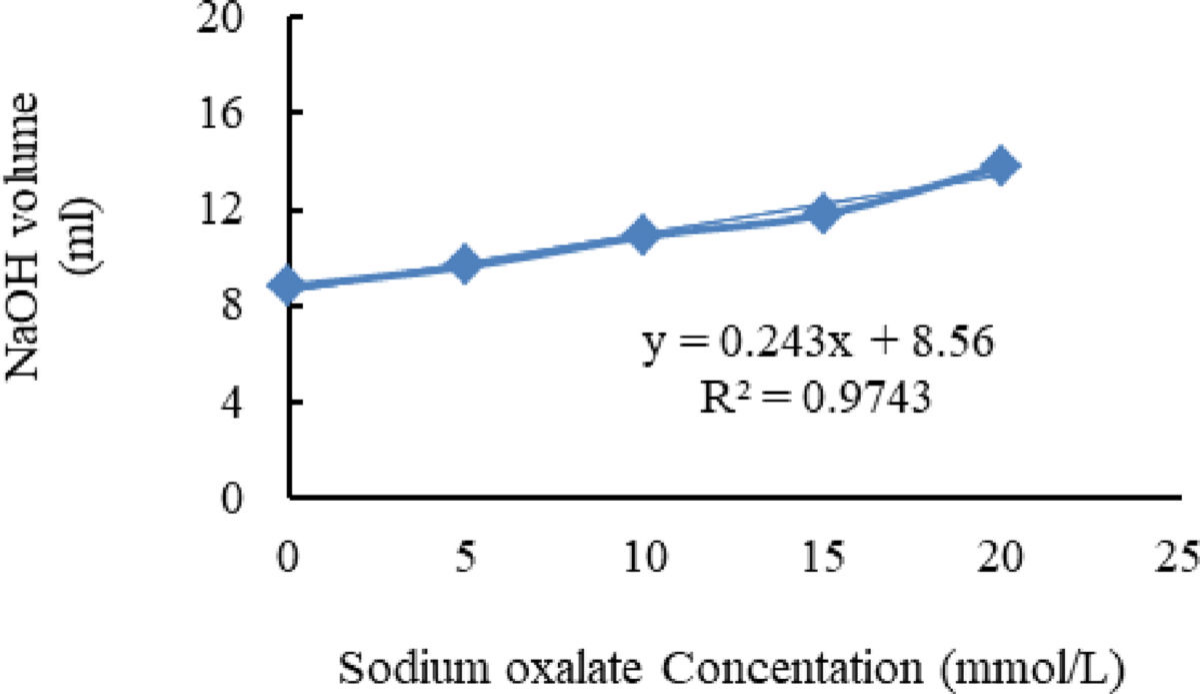
Calibration curves of sodium oxalate concentration.

**Figure 5.**
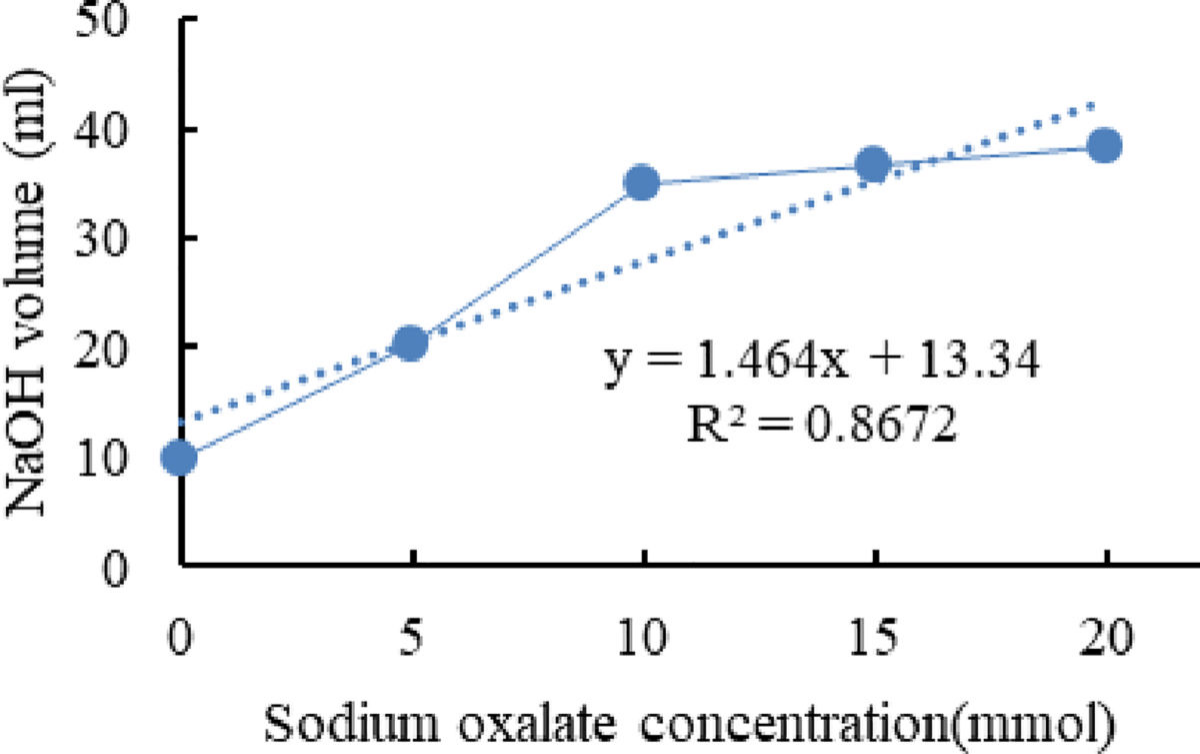
Calibration curves of sodium oxalate concentration and volumetric titration with NaOH after inoculation with *L. acidophilus*.

**Figure 6.**
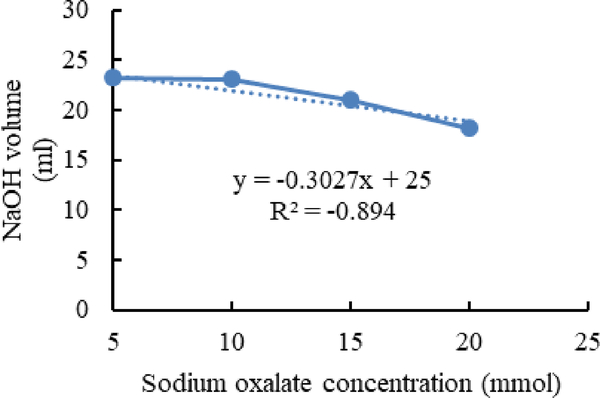
Calibration curves of sodium oxalate concentration and volumetric titration with NaOH after inoculation with *L.plantarum*.

Figure 4–6 show calibration curves of sodium oxalate concentration and volumetric titration with NaOH before inoculation, after inoculation with *L. acidophilus*, and after inoculation with *L. plantarum*, respectively.

### 3.2. Enzymatic method (Oxalate Assay kit)

**Figure 7.**
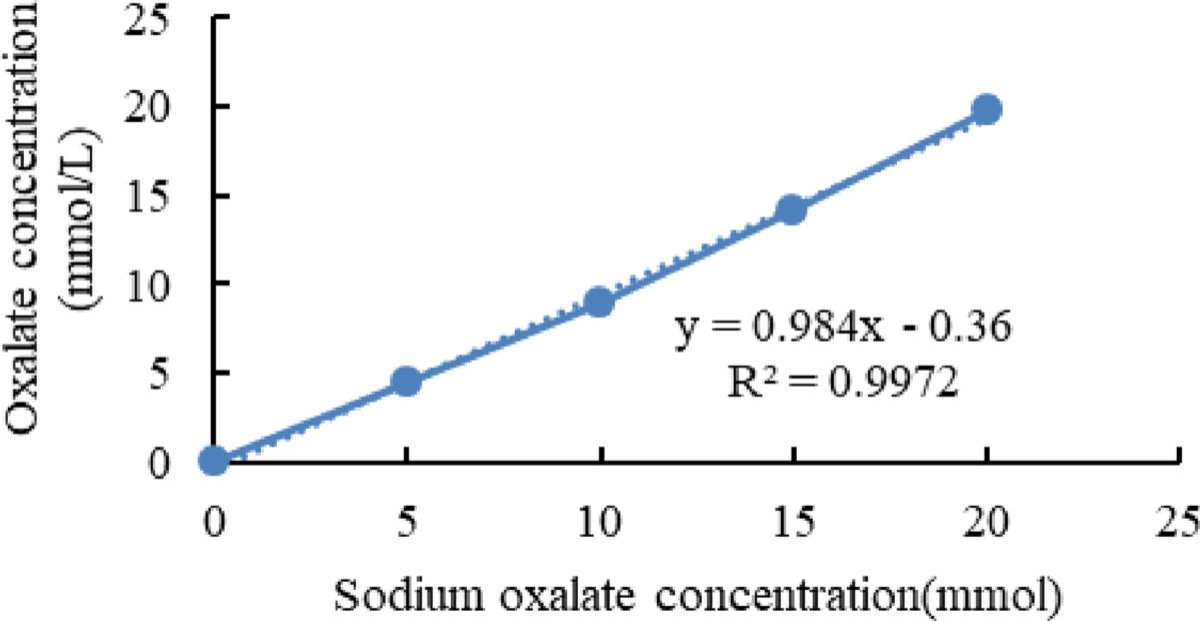
Calibration curves of sodium oxalate concentration and oxalate concentration before inoculation.

**Figure 8.**
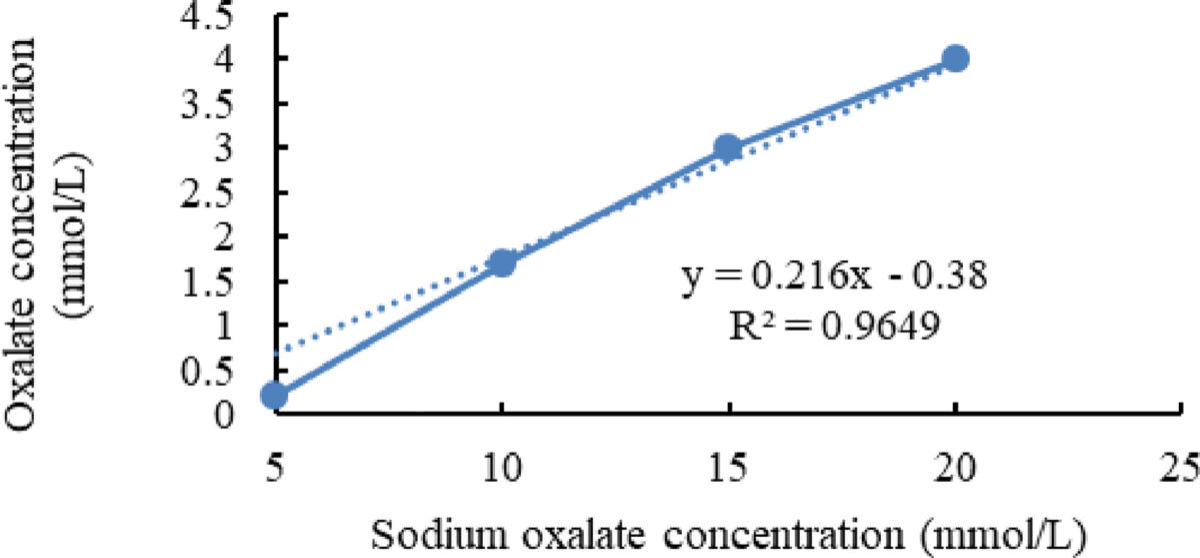
Calibration curves of sodium oxalate concentration and oxalate concentration after inoculation with *L. acidophilus*.

**Figure 9.**
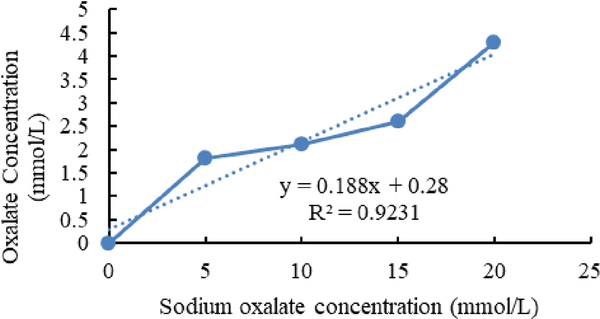
Calibration curves of sodium oxalate concentration and oxalate concentration after inoculation with *L. plantarum*.

Figure 7–9 shows calibration curves of sodium oxalate concentration and oxalate concentration before inoculation, after inoculation with *L. acidophilus*, and after inoculation with *L. plantarum*, respectively.

In figure 5 a sudden decline is seen in the acid detection via NaOH titration. It can be concluded that L. acidophilus couldn’t degrade sodium oxalate after 10 mmol/L concentration in the media.

In figure 6 the amount of acid detection increasing mildly. It shows that L. plantarum degraded sodium oxalate and convert it to oxalic acid which was detected via NaOH titration.

The enzymatic method appears to be the method of choice over titration methods for estimating the oxalate content of foods with a medium (0.05mmol/L) to high oxalate content (100 mmol/L) due to a faster analysis time. Accurate estimates of the oxalate content of foods should permit the role of dietary oxalate in urinary oxalate excretion and stone formation to be clarified. Other factors, apart from the amount of oxalate ingested, appear to exert a major influence over the amount of oxalate excreted in the urine. A new analytical method for oxalate content estimation is high-performance liquid chromatography (HPLC) method [31] which estimates oxalate from biological matrices without pre analysis sample preparation. But this method has a high cost and solvent price and this is the limitation which we ignore the use of this method in this research.

## 4. CONCLUSIONS

In titration with Potassium permanganate, the amount of permanganate is equal to sodium oxalate in the media. Before inoculation, there is no biodegradation in the media so the volume of permaganate which is used for balance titration point is higher than after inoculation with bacteria. The limitation of this method for oxalate content determination is that in this method we only can estimate the amount of sodium oxalate which is equilibrated with Potassium permanganate. For oxalate determination additional methods and calculation will be needed, therefore this method would be time and ingredient consuming.

In titration with NaOH, the amount of Sodium Hydroxide is equal to oxalic acid and other acids which are present in the media. In this experiment after inoculation with bacteria in a sudden decrease in the amount of NaOH in the titration process have been detected.

In the enzymatic method, the exact amount of oxalate has been detected and the data which is derived from this method only shows the amount of biodegraded oxalate in the media [16].

As shown in figure 2 and figure 3, in media inoculated with *L. acidophilus* more Sodium oxalate in compare with media inoculated with *L. plantarum* can detect. It is shown that *L. plantarum* can degrade more sodium oxalate than *L. acidophilus*.

## Figures and Tables

**Table 1.** Contents of vials for titration by potassium permanganate, NaOH, and enzymatic assay.

Sodium oxalate cocentration (mmol/L)	Before inoculation	After inoculation with *L. acidophilu L. plantarum*	

	**Permangenate volume (ml)**		

0	11.35	2.8	8

5	12.5	4.1	8

10	15	7	10

15	15.5	8.5	10.5

20	17.5	10	10.7

**NaOH volume (ml)**			

0	8.8	9.8	9.8

5	9.65	23.2	20.3

10	10.9	23	35

15	11.8	21	36.5

20	13.8	18.1	38.3

**Oxalate concentration (mmol)**			

0	0	0	0

5	4.5	0.2	1.8

10	9	1.7	2.1

15	14.1	3	2.6

20	19.8	4	4.3

**Table 2.** Material supplied for Oxalate Assay kit.

Material	Content

**Buffer solution**	25 ml

**Lyophilized enzymatic reagent**	5 vials

**Active caracole**	12 g

**Standard oxalic acid(0.25 mmol)**	5 ml

**Dilution solution**	60 ml

**Lyophilized coloring reagent**	1 vial

**Table 3.** Sample preparation for oxalate assay kit.

Content of tube	B(μL)

**Sample**	-

**Standard solution**	-

**Distilled water**	50

**Coloring reagent**	1000

**Enzymatic solution**	250

## References

[1] Abratt VR; and Reid SJ Oxalate degrading bacteria of the human gut as probiotics in the management of kidney stone disease. Adv. Appl. Microbiol 2010, 72, 63–87, 10.1016/S0065-2164(10)72003-7. 20602988

[2] Ahmadi N; Khosravi-Darani K; Zarean-Shahraki S; Mortazavian M; Mashayekh SM Fed-Batch Fermentation of Propionic, Acetic and Lactic Acid Production. Orient. J. Chem 2015, 31, 581–590, 10.13005/oic/310174.

[3] Ahmadi N; Khosravi-Darani K; Mortazavian M An overview of the biotechnological production of propionic acid: From upstream to downstream processes. Electron. J. Biotechnol 2017, 28, 67–75, 10.1016/i.eibt.2017.04.004.

[4] Ahmadi N; Khosravi-Darani K; Mohammad MA; Mashayekh SM Effects of Process Variables on Fed-Batch Production of Propionic Acid. J. Food Process. Preserv 2017, 41, 2, e12853, 10.1111/ifpp.12853.

[5] Attalla K; De S; Monga M Oxalate Content of Food: A Tangled Web. UROL 2014, 84, 555–560, 10.1016/i.iuro.2014.02.2274. 25168533

[6] Baker CJA The determination of oxalates in fresh plant material. Analyst 1952, 916, 304–309, 10.1039/an9527700340.

[7] Campieri C; Campieri M; Bertuzzi V; Swennen E; Matteuzzi D; Stefoni S; Pirovano F; Centi C Reduction of oxaluria after an oral course of lactic acid bacteria at high concentration. Kidney Int. 2001, 60, 10.1046/i.1523-1755.2001.0600031097.x. 11532105

[8] Duncan SH; Richardson AJ; Kaul P; Holmes RP; Allison MJ; Stewart CS Oxalobacter formigenes and its potential role in human health. Appl. Environ. Microb. 2002, 68, 3841–3847, 10.1128/AEM.68.8.3841-3847.2002. PMC124017 12147479

[9] Ghosh Das S; Savage GP; Oxalate content of Indian spinach dishes cooked in a wok. J. Food Compos, Anal 2013, 30, 125–129, 10.1016/nfca.2013.03.001.

[10] Holmes RP; Kennedy M Estimation of the oxalate content of foods and daily oxalate intake. Kidney Int. 2000, 57, 1662–1667, 10.1046/i.1523-1755.2000.00010.x. 10760101

[11] Kargar M; Afkari R; Ghorbani-Dalini S; Oxalate-degrading capacities of gastrointestinal lactic acid bacteria and urinary tract stone formation, Zahedan. J. Res. Med. Sci 2013, 15, 54–58.

[12] Khosravi DK; Zoghi A; Fatemi SSA Application of Plackett Burman design for citric acid production from pretreated and untreated wheat straw. Iran. J. Chem. Chem. Eng 2008, 27, 91–104.

[13] Lange JN; MufarriJ PW; Easter L; Knight J; Holmes RP; Assimos DG Fish Oil Supplementation and Urinary Oxalate Excretion in Normal Subiects on a Low-oxalate Diet. UROL. 2014, 84, 779–782, 10.1016/i.urology.2014.04.052. 25102784 PMC4243483

[14] Miller AW; Oakeson KF; Dale C; Dearing MD Microbial community transplant results in increased and long term oxalate degradation. Microb Ecol. 2016, 72, 470–478, 10.1007/s00248-016-0800-2. 27312892 PMC5155304

[15] Ohkawa H; Gas Chromatographic Determination of Oxalic Acid in Foods. J Assoc. Off Anal Chem, 1985, 68, 108–111. 3980399

[16] Ohlweiler AOM; Schneider MH Standardization of potassium permanganate by titration of sodium oxalate in presence of perchloric acid and manganese(II) sulfate, Analytica Chimica Acta 1972, 58, 477–480, 10.1016/S0003-2670(72)80031-X.

[17] Rokade K; Gote N; Sutar R; Biodegradation of calcium oxalate by newly isolated bacterial culture. J chem Pharmaceut Res 2015, 7, 179–182.

[18] Ruan QY; Zheng XQ; Chen BL; Xiao Y; Peng XX Determination of total oxalate contents of a great variety of foods commonly available in Southern China using an oxalate oxidase prepared from wheat bran. J. Food Compos. Anal 2013, 32, 11, 10.1016/i.ifca.2013.08.002.

[19] Siener R; Seidler A; Voss S; Hesse A The oxalate content of fruit and vegetable iuices, nectars and drinks. J. Food Compos. Anal 2016, 45, 108–112, 10.1016/i.ifca.2015.10.004.

[20] Trevaskis M; Trenerry V An investigation into the determination of oxalic acid in vegetables by capillary electrophoresis. Food Chem. 1996, 57, 323–330, 10.1016/0308-8146(95)00228-6.

[21] Von Burg R Oxalic acid and sodium oxalate. J Appl Toxicol. 1994, 14, 233–237. 8083486 10.1002/jat.2550140315

[22] Dodoo CC; Wang J; Basit AW; Stapleton PG Targeted delivery of probiotics to enhance gastrointestinal stability and intestinal colonization. Int J Pharm. 2017, 530, 224–229, 10.1016/nipharm.2017.07.068. 28764983

[23] Hatch M Gut microbiota and oxalate homeostasis. Annals of Translational Medicine 2017, 5, 36–36. 10.21037/atm.2016.12.70. 28217701 PMC5300851

[24] Onal EM; Afsar B; Covic A; Vaziri N; Kanbay M Gut microbiota and inflammation in chronic kidney disease and their roles in the development of cardiovascular disease. Hypertension Research 2018, 42, 123–140, 10.1038/s41440-018-0144-z. 30504819

[25] Thongprayoon C; Kaewput WT; Hatch S; Bathini T; Sharma K; Wyarnpreecha K; Ungprasert P; D’Costa M; Mao M; Cheungpasitporn W Effects of Probiotics on Inflammation and Uremic Toxins Among Patients on Dialysis: A Systematic Review and Meta-Analysis. Digestive Diseases and Sciences 2018, 64, 469–479, 10.1007/s10620-018-5243-9. 30099652

[26] Lieske JC Probiotics for prevention of urinary stones, Ann Transl Med. 2017, 5, 29–33, 10.21037/atm.2016.11.86. 28217694 PMC5300857

[27] Sönmez Ş; Önal DD; Beyatli Y Determination of the relationship between oxalate degradation and exopolysaccharide production by different Lactobacillus probiotic strains. Int J Dairy Technol. 2018, 71, 741–752, 10.1111/1471-0307.12513.

[28] Boonpheng B; Cheungpasitporn W; Wyarnpreecha K Renal disease in patients with celiac disease, a review. Minerva medica 2017, 109, 126–140, 10.23736/S0026-4806.17.05403-9. 28974086

[29] Arvans D; Jung YC; Antonopoulos D; Koval J; Granja I; Bashir M; Karrar E; Roy-Chowdhury J; Musch M; Asplin J Oxalobacter formigenes-derived bioactive factors stimulate oxalate transport by intestinal epithelial cells. J Am Soc Nephrol. 2017, 28, 876–87, 10.1681/ASN.2016020132. 27738124 PMC5328155

[30] Hoppe B; Niaudet P; Salomon R; Harambat J; Hulton SA; Van’t Hoff W; Moochhala SH; Deschenes G; Lindner E; Siogren A; Cochat P ARandomised phase I/II trial to evaluate the efficacy and safety of orally administered Oxalobacter formigenes to treat primary hyperoxaluria. Pediatr Nephrol 2017, 32, 781–90, 10.1007/s00467-016-3553-8. 27924398

[31] Siener R; Seidler A; Voss R; Hesse A Oxalate content of beverages. Journal of Food Composition and Analysis 2017, 63,184–188, 10.1016/i.ifca.2017.08.005.

[32] Zhao C; Yang H; Zhu X; Li Y; Wang N; Han S; Xu H; Chen Z; Ye Z Oxalate-Degrading Enzyme Recombined Lactic Acid Bacteria Strains Reduce Hyperoxaluria. Urology 2018, 113, 1–7, 10.1016/i.urology.2017.11.038. 29198849

[33] Sadaf H; Raza SI; Hassan SW; Role of gut microbiota against calcium oxalate. Microbial Pathogenesis 2017, 109, 287–291, 10.1016/i.micpath.2017.06.009. 28624518

[34] Assimos DG Re: Understanding the Gut-Kidney Axis in Nephrolithiasis: An Analysis of the Gut Microbiota Composition and Functionality of Stone Formers. The Journal of Urology, 2018, 200, 940–941, 10.1136/gutinl-2017-315734. 30360336

[35] Mahmoodpoor F; Rahbar SY; Barzegari A; Ardalan MR; Zununi VS, The impact of gut microbiota on kidney function and pathogenesis. Biomedicine & Pharmacotherapy 2017, 93, 412–419, 10.1016/i.biopha.2017.06.066. 28654798

[36] Nguyễn HVH; Lê MH; Savage PG Effects of maturity at harvesting and primary processing of cocoa beans on oxalate contents of cocoa powder. Journal of Food Composition and Analysis 2018, 67, 86–90, 10.1016/i.ifca.2018.01.007.

